# Endoscopic ultrasonography for pre-operative local assessment and endoscopic ultrasonography-guided marking before gastrojejunostomy for duodenal obstruction using magnetic compression anastomosis

**Published:** 2021-09-20

**Authors:** Hideaki Kawabata, Kojiro Nakase, Yuji Okazaki, Tetsuya Yamamoto, Katsutoshi Yamaguchi, Yuki Ueda, Masatoshi Miyata, Shigehiro Motoi

**Affiliations:** Department of Gastroenterology, Kyoto Okamoto Memorial Hospital, Japan

**Keywords:** magnetic compression anastomosis, endoscopic ultrasonography, gastrojejunostomy, endoscopy, duodenal obstruction

## Abstract

**Background and Aim:**

A 93-year-old woman who was bedridden with severe dementia was referred to our department with a 3-day history of repeated vomiting after meals. Computed tomography revealed significant dilatation of the duodenum up to the level of the third portion, which was compressed by a large, low-density mass. Upper gastrointestinal endoscopy showed narrowing of the third portion of the duodenum with edematous mucosa covered with multiple white spots, where the endoscope was able to pass through with mild resistance. B-cell lymphoma was histopathologically suspected from biopsy specimens of the mucosa. We performed gastrojejunostomy through the magnetic compression anastomosis (MCA) technique. We prepared two neodymium magnets: Flat plate shaped (15 × 3 mm) with a small hole 3 mm in diameter; a nylon thread was passed through each hole. We then confirmed the absence of no non-target tissue, including large vessels and intestine adjacent to the anastomosis where the magnets were to be placed using endoscopic ultrasonography (EUS) from the stomach. EUS-guided marking using biopsy forceps by biting the mucosa and placing a hemoclip was performed at the anastomosis site in the stomach. The magnet was pushed and delivered to the duodeno-jejuno junction, and another magnet was delivered to the marking point in the stomach. The magnets were attracted toward each other transmurally. The magnets fell into the colon by 11 days after starting the compression, and the completion of gastrojejunostomy was confirmed.

**Relevance for Patients:**

Endoscopic gastrojejunostomy using MCA is useful as a minimally invasive alternative treatment for duodenal obstruction. EUS for the pre-operative local assessment and EUS-guided marking can ensure the safety of the MCA procedure.

## 1. Introduction

Magnetic compression anastomosis (MCA) was developed as a low invasive, alternative treatment for enteric or biliary obstruction [1-3] that can also endoscopically create well-formed anastomoses without surgical assistance [4-8]. However, a few post-operative complications, such as anastomotic leakage, damage to unintended tissue between the mated magnets and bleeding have been reported [1,7]. Pre-operative examinations, including gastrointestinal (GI) series and enhanced (three dimensional) computed tomography (CT), have been performed to assess the local condition of the anastomotic sites and calculate the distance between the magnets [1,4,6,7]. However, the local condition at the anastomotic sites, especially in case of enteric anastomosis, can be easily influenced and malleable due to enteric peristalsis, the GI content, such as air, juice and stool retention, and the insertion of the endoscope, which may induce unintended tissue pinching.

Developed to mitigate the above-mentioned issues, endoscopic ultrasonography (EUS) provides high-resolution, real-time imaging of the GI tract and surrounding extramural structures [9,10]. In biliary MCA, contrast-enhanced EUS with color Doppler and intraductal US were reportedly used for the detection of major blood vessels and foreign bodies between the magnets [5,11]. EUS is also useful in EUS-guided gastrojejunostomy using lumen-apposing metal stents for the palliation of malignant gastric outlet obstruction [12,13].

We herein report a case of EUS performed for the pre-operative local assessment and EUS-guided marking before endoscopic gastrojejunostomy for duodenal obstruction using MCA.

## 2. Case Report

**Figure 1:**
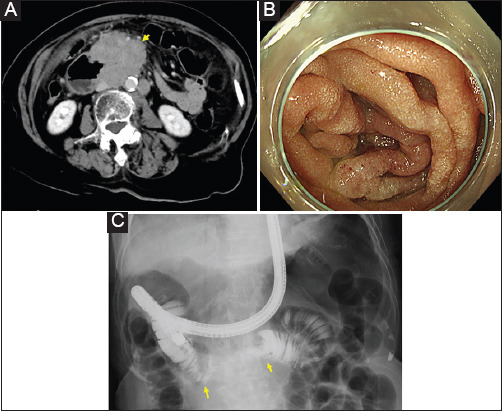
Enhanced abdominal CT revealed a severely dilated stomach and significant dilatation of the duodenum up to the level of the third portion, which was compressed by a large, low-density mass, suggestive of malignant lymphoma (arrow) (A). Upper gastrointestinal endoscopy showed narrowing of the third portion of the duodenum with edematous mucosa covered with multiple white spots, where the endoscope was able to pass through with mild resistance (B). Fluoroscopy showed severe stricture at the third portion of the duodenum (arrows) (C).

A 93-year-old woman who was bedridden with severe dementia was referred to our department in April 2021 with a 3-day history of repeated vomiting after meals. She had no significant history of abdominal surgical issues. An examination revealed abdominal distension without tenderness. Enhanced abdominal CT revealed a severely dilated stomach and significant dilatation of the duodenum up to the level of the third portion, which was compressed by a large, low-density mass, suggestive of malignant lymphoma (Figure 1A).

Upper GI endoscopy showed narrowing of the third portion of the duodenum with edematous mucosa covered with multiple white spots, where the endoscope was able to pass through with mild resistance (Figure 1B). B-cell lymphoma was histopathologically suspected from biopsy specimens of the mucosa. Fluoroscopy showed severe stricture at the third portion of the duodenum (Figure 1C).

We performed gastrojejunostomy through the MCA technique because her situation was not improved despite conservative therapy, including liquid food and postural change of 1-week duration, and she could not tolerate aggressive treatment, including surgery, radiotherapy, and chemotherapy, due to her age, general condition, and severe dementia.

Furthermore, stent placement for palliation of duodenal obstruction carried a risk of stent dislocation because of compressive stricture. Written informed consent was obtained from the patient’s family, and this procedure was approved by the ethics committee of our institute (2019-37).

**Figure 2:**
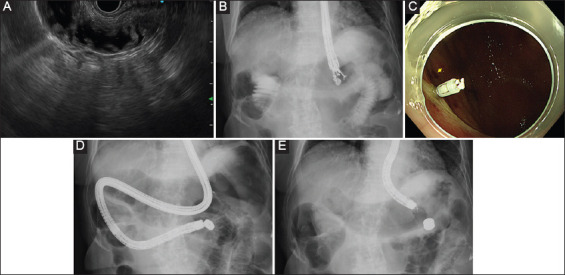
EUS with color Doppler from the stomach revealed the absence of non-target tissue, including small intestine, colon, and large vessels, adjacent to the anastomosis where the magnets were to be placed (A). EUS-guided marking using biopsy forceps by biting the mucosa (arrows) and placing a hemoclip was performed at the anastomosis site in the stomach (B, C). A flat plate-shaped magnet was pushed and delivered to the duodeno-jejuno junction adjacent to the marking hemoclip as an indicator by a forward-viewing endoscope (D). Another flat plate-shaped magnet was delivered endoscopically to the marking point in the stomach using biopsy forceps. The magnets were attracted toward each other transmurally (E).

**Figure 3:**
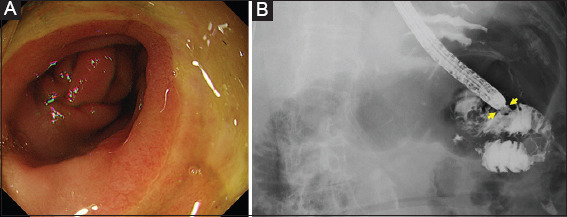
The completion of gastrojejunostomy was confirmed 11 days after starting the compression (arrows) (A, B).

We prepared two neodymium magnets: Flat plate shaped (15 × 3 mm; Magfine, Sendai, Japan) with a small hole 3 mm in diameter; a nylon thread was passed through each hole. As a spasmolytic, glucagon (0.5 mg, Glucagon G Novo; Novo Nordisk Pharma Ltd, Tokyo, Japan) was intravenously administered. We then confirmed the absence of no non-target tissue, including small intestine, colon, and large vessels, adjacent to the anastomosis where the magnets were to be placed using EUS (GF-UCT260; Olympus Medical Systems, Tokyo, Japan) from the stomach, which had been filled with water at the anal side of the duodenal stricture (Figure 2A). EUS-guided marking using biopsy forceps by biting the mucosa (Figure 2B) and placing a hemoclip (EZ clip; Olympus Medical Systems, Tokyo, Japan) was performed at the anastomosis site in the stomach (Figure 2C). The magnet was then pushed and delivered to the duodeno-jejuno junction adjacent to the marking hemoclip as an indicator by a forward-viewing endoscope (Figure 2D). Subsequently, another magnet was delivered endoscopically to the marking point in the stomach using biopsy forceps. The magnets were then allowed to be attracted toward each other transmurally (Figure 2E). The magnets fell into the colon by 11 days after starting the compression, and the completion of gastrojejunostomy was confirmed (Figure 3A, 3B). The patient subsequently started eating a normal diet and remained asymptomatic until being transferred from the hospital 1 month after the procedure.

## 3. Discussion

Endoscopic gastrojejunostomy using MCA was useful as a minimally invasive treatment for duodenal obstruction. Animal studies have proven the feasibility and safety of MCA, which can consistently create histologically well-formed anastomoses with a burst strength comparable to or better than that of handsewn or stapled anastomoses [14-16]. We found two clinical studies investigating the utility of enteroenterostomy using MCA without additional anastomotic stent placement [1,4]. Yamanouchi *et al*. [1] reported that 43 of 45 patients successfully achieved endoscopic enteroenteric MCA with just two complications of anastomotic leakage and damage to unintended tissue between the mated magnets as well as anastomotic stenosis in 20 – 25% of patients. Kamada *et al*. [4] showed that technical success of GI MCA was achieved in all 14 patients, although late complication with anastomotic stenosis occurred in two cases. Follow-up esophagogastroduodenoscopy was not performed in our present patient as she remained asymptomatic after starting meal intake and had risk factors associated with invasive procedures, including EGD. Thus, endoscopic enteroenterostomy using MCA can be a good alternative treatment in patients with GI obstruction for whom aggressive treatment carries a high risk and is contraindicated for palliative stent placement, such as cases of benign or compressive stricture. However, endoscopic MCA involves passing a magnet with an endoscope through the stricture site, with balloon dilatation able to be used if a guidewire is passed through the stricture site [6].

More studies with a larger population and prophylactic measures to prevent post-operative anastomotic stenosis are needed to confirm the utility and safety of this procedure for its general use in the clinical setting.

Recently, the concept of EUS-guided gastroenterostomy using lumen-apposing metal stents for the management of gastric outlet obstruction has been proposed, and favorable outcomes have been reported [12,17]. A systematic review and meta-analysis [17] demonstrated a pooled technical success rate of 92% (95% confidence interval [CI]: 88 – 95%) and clinical success rate of 90% (95% CI: 85 – 94%) with fewer adverse events and lower recurrence rate than surgical gastrojejunostomy or enteral stenting. However, this technique requires technical expertise and costly devices, and stenting associated adverse events, including stent misdeployment, gastric leak, and peritonitis, can occur, leading to life-threating serious conditions requiring surgical salvation.

EUS for the pre-operative local assessment and EUS-guided marking can ensure the safety of MCA. A local assessment followed by optimal positioning of magnets is crucial in enteric MCA. Pinching of unintended tissue, including large vessels and intestine, by mated magnets may cause massive bleeding and ileus, respectively, which can require surgical treatment in the worst cases. EUS with color Doppler from the stomach can easily visualize the real-time, local condition, including that of the vessels and intestine at the anastomosis site, calculate the distance between the magnets, and be adjusted to determine the optimal position. Furthermore, marking with mucosal biting using biopsy forceps and a hemoclip enables each magnet to be accurately delivered and placed at the intended position for anastomosis. However, hemoclips made of stainless steel should be used to prevent attraction to the magnets. This is the first report introducing pre-operative EUS-guided marking in gastrojejunostomy using MCA.

## 4. Conclusion

In conclusion, endoscopic gastrojejunostomy using MCA is useful as a minimally invasive alternative treatment for duodenal obstruction. EUS for the pre-operative local assessment and EUS-guided marking can ensure the safety of the MCA procedure. More studies with larger populations and prophylactic measures for preventing post-operative anastomotic stenosis are needed for the general use of this procedure in the clinical setting.

### Conflicts of Interest

The authors declare that they have no conflicts of interest.

### Informed Consent

Written informed consent was obtained from the patient.
